# Environmental and anthropogenic factors affecting the probability of occurrence of *Oncomegas wageneri* (Cestoda: Trypanorhyncha) in the southern Gulf of Mexico

**DOI:** 10.1186/s13071-015-1222-6

**Published:** 2015-11-26

**Authors:** Víctor M. Vidal-Martínez, Edgar Torres-Irineo, David Romero, Gerardo Gold-Bouchot, Enrique Martínez-Meyer, David Valdés-Lozano, M. Leopoldina Aguirre-Macedo

**Affiliations:** Laboratorio de Parasitología, Centro de Investigación y de Estudios Avanzados del Instituto Politécnico Nacional, Unidad Mérida, Km 6 Carretera Antigua a Progreso, Cordemex, Mérida, Yucatán 97310 México; Laboratorio de Tecnologías Geoespaciales, Centro de Investigación y de Estudios Avanzados del Instituto Politécnico Nacional, Unidad Mérida, Km 6 Carretera Antigua a Progreso, Cordemex, Mérida, Yucatán 97310 México; Posgrado de Geografía. Facultad de Filosofía y Letras, Universidad Nacional Autónoma de México, Circuito Interior, Ciudad Universitaria, 04510 México DF México; Oceanography Department and GERG, Texas A&M University, College Station, TX USA; Laboratorio de Análisis Espaciales, Dpto. Zoología, Instituto de Biología, Universidad Nacional Autónoma de México, Apdo. Postal 70-153, 04510 México, DF México; Centro de Investigación y de Estudios Avanzados del Instituto Politécnico Nacional, Unidad Mérida, Km 6 Carretera Antigua a Progreso, Cordemex, Mérida, Yucatán 97310 México

**Keywords:** Cestoda, Trypanorhyncha, Bioindicators, Environmental impact, Flatfish, Contamination, Gulf of Mexico

## Abstract

**Background:**

Understanding the environmental and anthropogenic factors influencing the probability of occurrence of the marine parasitic species is fundamental for determining the circumstances under which they can act as bioindicators of environmental impact. The aim of this study was to determine whether physicochemical variables, polyaromatic hydrocarbons or sewage discharge affect the probability of occurrence of the larval cestode *Oncomegas wageneri,* which infects the shoal flounder, *Syacium gunteri*, in the southern Gulf of Mexico.

**Methods:**

The study area included 162 sampling sites in the southern Gulf of Mexico and covered 288,205 km^2^, where the benthic sediments, water and the shoal flounder individuals were collected. We used the boosted generalised additive models (boosted GAM) and the MaxEnt to examine the potential statistical relationships between the environmental variables (nutrients, contaminants and physicochemical variables from the water and sediments) and the probability of the occurrence of this parasite. The models were calibrated using all of the sampling sites (full area) with and without parasite occurrences (n = 162) and a polygon area that included sampling sites with a depth of 1500 m or less (n = 134).

**Results:**

*Oncomegas wageneri* occurred at 29/162 sampling sites. The boosted GAM for the full area and the polygon area accurately predicted the probability of the occurrence of *O. wageneri* in the study area. By contrast, poor probabilities of occurrence were obtained with the MaxEnt models for the same areas. The variables with the highest frequencies of appearance in the models (proxies for the explained variability) were the polyaromatic hydrocarbons of high molecular weight (PAHH, 95 %), followed by a combination of nutrients, spatial variables and polyaromatic hydrocarbons of low molecular weight (PAHL, 5 %).

**Conclusions:**

The contribution of the PAHH to the variability was explained by the fact that these compounds, together with N and P, are carried by rivers that discharge into the ocean, which enhances the growth of hydrocarbonoclastic bacteria and the productivity and number of the intermediate hosts. Our results suggest that sites with PAHL/PAHH ratio values up to 1.89 promote transmission based on the high values of the prevalence of *O. wageneri* in the study area. In contrast, PAHL/PAHH ratio values ≥ 1.90 can be considered harmful for the transmission stages of *O. wageneri* and its hosts (copepods, shrimps and shoal flounders). Overall, the results indicate that the PAHHs affect the probability of occurrence of this helminth parasite in the southern Gulf of Mexico.

**Electronic supplementary material:**

The online version of this article (doi:10.1186/s13071-015-1222-6) contains supplementary material, which is available to authorized users.

## Background

Understanding the environmental factors influencing the probability of occurrence of parasitic species is fundamental for determining the circumstances under which they can be affected by human activities. This knowledge is necessary because parasites can be useful as bioindicators, i.e., species or communities that are used to assess the quality of the marine environment and how it changes over time [1–4].

Marine biologists traditionally have used free-living, benthic organisms as bioindicators to assess the environmental quality of the oceanic sediments associated with anthropogenic impacts [5–9]. However, there are limitations to the use of benthic, free-living organisms as bioindicators, such as the following: 1) the large number of samples (and funding) required for quantitative sampling; 2) seasonal variation affecting the distribution and abundance of the organisms in addition to the water or the sediment quality; 3) many methods (and indices) are available for the analyses, suggesting a great heterogeneity in the interpretation of the results; and 4) a limited taxonomic knowledge regarding the various groups because of the large number of species in the benthic realm [10–12].

The parasites of aquatic organisms are also affected by anthropogenic and natural environmental influences and have been proposed as alternative bioindicators [2, 4]. There are several advantages to the use of parasites as bioindicators of environmental quality: 1) their communities are far less speciose than those of free-living, benthic organisms; 2) their taxonomy and life cycles are relatively well known; and 3) from a parasite point of view, each individual host is an island (or habitat), and statistically, each host becomes a “sampling unit” with its own set of parasite species [2, 4, 13–18]. At this point, parasites are accepted as an integral part of the environment and suffer the same type of influence from natural and anthropogenic variables on their various life stages (e.g., transmission forms, such as cercariae or coracidia; larval stages, such as metacercariae or cystacanths; and adult stages) [3, 19, 20]. However, even though it has been possible on a small spatial scale for the freshwater or coastal environments to determine links between the environmental variables affecting the ecological metrics (e.g., species number or relative abundance) of parasites (e.g., [21–23]), the knowledge regarding the environmental factors influencing the probability of occurrence of the parasites at a seascape scale (hectares to thousands of square km^2^) is almost lacking. Thus, an important step required to integrate parasites as bioindicators of environmental quality in the oceans at a seascape scale is to determine the environmental or anthropic factors that affect the parasite community or the population metrics.

Ecological niche models (ENMs) are useful for providing insight into the mechanisms by which environmental variables influence the probability of occurrence of both terrestrial and marine organisms [24, 25]. Most of these models are correlative in nature and determine whether there are statistical associations between environmental, biological and/or geographical variables and species abundances or occurrences to establish the sets of conditions under which the species can maintain viable populations [26, 27]. Although ENMs have been profusely used in the terrestrial realm to address a myriad of topics (e.g., [28–30]), these models have a more recent history in the marine environment (e.g., [31, 32]).

During studies in the southern Gulf of Mexico to determine the environmental quality regarding the sediments, water and organisms for the Mexican oil company (PEMEX), we obtained data on the geographical distribution and abundance of the helminth parasites infecting the shoal flounder, *Syacium gunteri*. These studies were developed for PEMEX because inland and offshore oil extraction, together with agriculture, are significant economic activities in the southern Gulf of Mexico, and high concentrations of nutrients from the river runoff, polycyclic aromatic hydrocarbons, and other contaminants, such as pesticides, are released into the environment (e.g., [33, 34]). Thus, to study the potential effects of natural environmental factors (e.g., oxygen concentration and salinity), nutrients and chemical pollutants on the probability of occurrence of parasites, we selected the larval cestode, *Oncomegas wageneri*, because of its high overall prevalence in the study area (84 %). The life cycle of *O. wageneri* is unknown. However, Palm (1995) [35] described that *O. wageneri* occupy a special position within the Eutetrarhynchoidea because its plerocercoid stage lacks a blastocyst. Thus, based on the life cycle of an eutetrarhynchid relative (*Prochristianella hispida*) [36], *O. wageneri* should have copepods and shrimp as first and second intermediate hosts, respectively, while stingrays should be the definitive hosts. In this case, the shoal flounder could act as a paratenic host or as a potential third intermediate host [Dr. Bjoern Schaeffner, Universidade de São Paulo, pers. com.]. Because this parasite has transmission stages and intermediate hosts that are exposed to the environment, its infection parameters should reflect the environmental conditions experienced by all of these organisms. In this sense, the values of the infection parameters, such as the prevalence and mean abundance, can be considered indirect measures of the net colonisation of the fish (sensu [37]). However, even when the fish sampling procedure used was standardised, the number of fish collected was highly uneven among the sampling sites, making it difficult to compare the abundance of *O. wageneri* among the sampling sites. To overcome this problem, we transformed the parasite data from abundance values to presence/absence values. This method was adapted from the field of community ecology, in which estimators of the number of species are frequently biased by the number of units sampled at a specific locality and in which presence/absence descriptors perform well (e.g.,[38, 39]). This method allowed us to use a binomial distribution for all the ENMs to estimate the probability of *O. wageneri* occurrence at the sampling site level based on the potential effect of different environmental and anthropic variables (i.e., pollutants). Thus, the objective of the present paper was to determine whether the probability of the occurrence of *O. wageneri* presented statistical associations with natural physicochemical environmental variables, nutrients and polycyclic aromatic hydrocarbons at the seascape level in the southern Gulf of Mexico.

## Methods

### Study area and sampling procedures for sediments and water

**Fig. 1:**
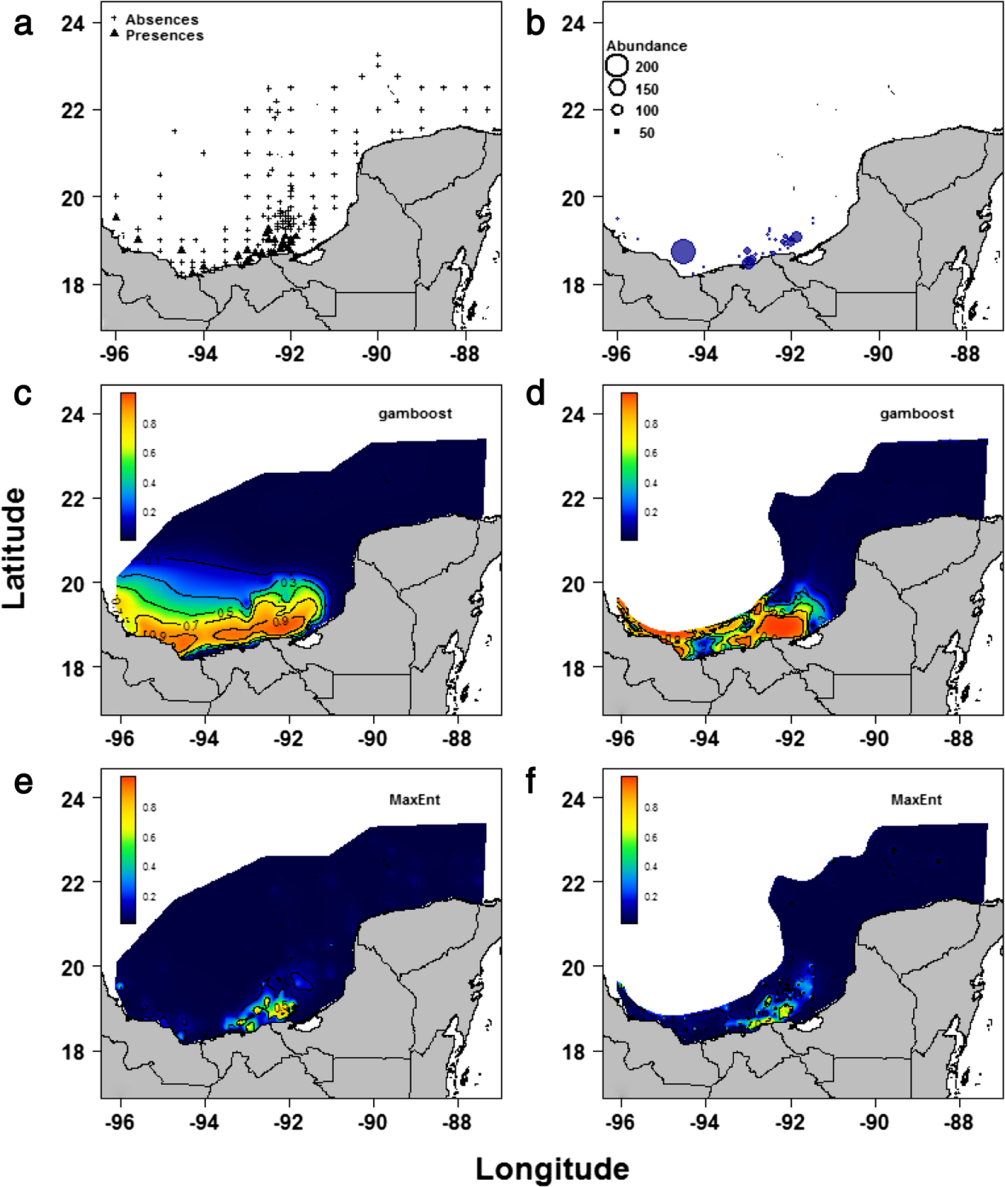
Geographical distribution of the larval cestode *Oncomegas wageneri* in the southern Gulf of Mexico. **a** Presence (▲) and absence (**+**) of *O. wageneri* at the 162 sampling sites of the oceanographic expedition Xcambo 2 between September and October 2005. **b** Total number of *O. wageneri* per sampling site. **c** Probability of occurrence of *O. wageneri* using a boosted generalised additive model for the full area (n = 162 sampling sites). **d** Probability of occurrence of *O. wageneri* using a boosted generalised additive model for the polygon area (n = 134 sampling sites at a depth of 1500 m or above). **e** Probability of occurrence of *O. wageneri* using the MaxEnt for the full area. **f** Probability of occurrence of *O. wageneri* using the MaxEnt for the polygon area

The study area included 162 sampling stations in the southern Gulf of Mexico (Fig. 1) and covered 288,205 km^2^. Bottom sediments were collected between September 6 and October 8, 2005, at water depths between 0.1 and 200 m with a 36 m long shrimp boat using a Smith-McIntyre grab with a weight of 80 kg and a volumetric capacity of 0.018 m^3^. For water depths between 201 and 3571 m, the bottom sediments were collected from the oceanographic vessel OV Justo Sierra with a 0.25 m^2^ Hessler Sandia MK-III box corer. Water samples were taken at 5 m depth using 1-gallon amber glass bottles that were closed under water to avoid contamination with surface mixtures. We obtained 32 physicochemical parameters from the water and the sediments, including oxygen (mg/L), salinity (UPS), pH, and nitrogen (micromol/g), among others (see Additional file 1: Table S1 in the online supporting material for a complete list). The hydrocarbon sampling procedures have been described elsewhere [34, 40, 41]. The sediment samples were placed in high-density polythene (HDPE) bags and kept at 4 °C for transport to CINVESTAV-IPN, Unidad Mérida. The types of hydrocarbons and metals and the physicochemical characteristics of the sediment were determined in the Geochemistry and the Marine Chemistry laboratories, respectively, using standardised methods [42–44]. Both the environmental and the biological data were obtained from all study sites within a very narrow window of time (within a month). Therefore, the temporal variability in the variables was assumed to be minimal, and most of the variability in the data was considered to be spatial.

### Sampling procedures for the flatfishes and helminth parasites

The shoal flounders for the present study were collected between September and October 2005 by professional fishermen based on their commercial fishing permit (issued by the Secretaria de Ganaderia, Desarrollo Rural, Pesca y Alimentación, number: 01067, and available upon request). The collection was conducted using a commercial fishing boat with 4 shrimp trawl nets with a light mesh of 1¾ inches by 1½ inches in the bag and with a turtle excluder device (Super Shooter). The trawls were performed by making circles around the sampling site for 50-60 min at a speed of 0.6-0.7 knots/h and were performed for the shortest period of time possible considering the welfare issues [45, 46]. Because shoal flounders are fragile animals [47], most of them were dead when the nets landed into the boat. The flounders that were still alive were kept in a container with marine water and an oxygen supply and later were euthanised with 100 mg/L of benzocaine until opercular movements ceased; posteriorly, the brain was then severed by spiking [48]. The fishing activities did not involve endangered or protected species according to Mexican regulations (NOM-059-SEMARNAT-2001).

The statement of ethics approval for the present paper was provided by the Institutional Animal Care and Use Committee (IACUC) from the Center of Research and Advanced Studies (Protocol number: 0138-15) and is available upon request.

The fish collected (n = 194) were kept in isolated plastic bags within cold storage coolers (-20 °C) on the vessel until they were transported to CINVESTAV-IPN Unidad Mérida for examination. Once in the laboratory, the total length, the standard length, the maximum height (cm) and weight (g) were recorded for each fish. Subsequently, a helminthological examination was performed to search for *O. wageneri*. The parasitological examinations were conducted using stereomicroscopes, and a 0.7 % saline solution was used as necessary. The abundance of *O. wageneri* was quantified by squashing the mesenteries and muscle fillets between two glass plates. The detected parasites were preliminarily identified in wet mounts prepared with ammonium picrate fixative on semipermanent slides [49, 50]. Additional *O. wageneri* individuals (usually 10-20) were fixed in 4 % formalin in labelled vials, stained with Mayer’s paracarmine, mounted in Canada balsam for subsequent taxonomic identification and identified at the species level using specialised literature (e.g., [51, 52]). In both cases (plerocerci stained with ammonium picrate or Mayer’s carmine), the identification of most individuals was based on the scolex measurements, the presence and measurements of the macrohook in the bothrial surface of the basal swelling of the tentacles, the number of hook rows in the basal armature, and the number of hooks in the basal and meta-basal armature of the tentacles. Because the morphological characteristics of all of our plerocerci were within the ranges indicated for *O. wageneri* (see [51]), we assumed that all of them belonged to this species. However because the plerocerci lack adult morphological characteristics, such as the distribution of testes posterior to the ovary, the potential presence of other species cannot be completely ruled out. The host species was identified by ichthyologists at the Necton Laboratory (CINVESTAV-IPN Mérida Unit).

Even when the fish sampling procedure used was standardised, the number of fish collected was highly variable among the sampling sites (Additional file 1: Table S1). For the sampling sites from which a fair number of fish were collected, no more than 10 fish were examined in the search for *O. wageneri*. However, no more than 1-5 individual fish were collected from several of the sampling sites. Clearly, this fact makes it very difficult to compare the abundance of the parasite species among the sampling sites because this parameter depends heavily on the number of fish caught. To overcome this problem, we transformed the parasite data from the mean abundance values to the presence/absence values. We divided the number of *O. wageneri* by the number of fish examined at each sampling site. Then, instead of using the mean abundance value, we used the value of this metric to represent the number of times the parasite was present. For example, if we caught 5 shoal flounders at a specific sampling site and found that they were infected with 5, 2, 3, 0 and 0 *O. wageneri*, this meant that we had 3 presences and 2 real absences at that sampling site. This method of changing abundance data to binary data (presences and absences) allowed us to overcome the problem of the dependence of *O. wageneri* abundance on the number of fish obtained per sampling site and to represent the number of parasites as the number of presences. This method was adapted from the field of community ecology, in which species richness estimators are also frequently biased by the number of sampling units considered at a specific locality, and the presence/absence descriptors perform well (e.g.,[38, 39]). The use of this methodology allowed us to choose a binomial distribution for the dependent variable in the generalised additive model (see below) to estimate the probability of the occurrence of *O. wageneri* in response to the environmental and pollution variables at the sampling site level. In addition to the 32 environmental variables recorded, to factor in the contribution of unknown environmental variables acting at different spatial scales into the analysis, we used a principal coordinates of neighbour matrices (PCNM) analysis to generate a set of spatial variables from the geographical position of each sampling site [53–56]. This set of spatial variables (called PCNM vectors) represents a spectral decomposition of the spatial relationships among the sites that corresponds to all of the spatial scales that can be perceived from the data [56]. The PCNM vectors were grouped into the following three spatial scales: large (58–22 km); medium (21–15 km) and small (14–2 km). These variables were used as independent variables, and the probability of the occurrence of *O. wageneri* per sampling site was used as the dependent variable in both the generalised additive models (boosted GAMs hereafter) and the maximum entropy algorithm (MaxEnt hereafter).

### Data analysis

#### Calibration of the Generalised Additive Models with Mboost

We fitted the generalised additive models (GAMs) using a boosting algorithm based on component-wise univariate base learners implemented in the R package mboost [57] to examine whether the environmental variables statistically related to the probability of the occurrence of *O. wageneri*. In the mboost package, a GAM has two parts: a distributional part (or assumption in Hofner et al. [57]) and a structural part. The distributional part specifies the conditional distribution of the dependent variable. In this case, we chose the binomial distribution because our data were transformed to parasite presence or absence. The structural part specifies how the predictors (our environmental, pollution and PCNM variables) are related to the dependent variable [57]. The mboost package is essentially a machine-learning algorithm and performs a variable selection during the fitting process [57, 58]. In the boosted GAM, the structure of each predictor is assumed to be additive, so many base-learners (similar to the classical smoothers in the GAMs [59]), such as penalised B-splines or P-splines, can be used. We used base-learners based on B-splines on the covariates (called bbs hereafter). The presence of spatial autocorrelation violates the assumption of observation independence and can bias the results [60, 61]. Because such a situation is common in ecological data [57], we used a spatial base-learner on the geographical coordinates that addresses the spatial autocorrelation in the boosted GAM [61]. Because we used a boosted GAM, the effects in the model were of two types: a global effect (related to the environmental variables) and a local effect (related to the spatial autocorrelation and the nonstationarity assumption), following the suggestions of Kneib et al. [62] and Hothorn et al. [63]. Thus, the boosted GAM was able to perform the following tasks: 1) fit a complex model considering all the available variables; 2) choose the most relevant variables (the ones providing the most information); 3) allocate the information to the local and global components of the model; and 4) divide the input data into a training set and a test set, which is one of the best ways to ensure that the results are not artefacts of overfitting. The boosted GAM implementations [57], as well as the PCNM analyses, are based on the R software for statistical computing [64].

### Calibration of MaxEnt

The maximum entropy algorithm (MaxEnt) estimates the probability of habitat suitability for the establishment of viable populations by finding a probability distribution that is closest to uniform but restricted by the mean values of the environmental variables at the sampling sites [65]. We concur with Merow et al. [66] in that using only the autofeatures of the MaxEnt could produce misleading results. Thus, we followed their recommendations as much as possible when choosing the MaxEnt settings. The following settings were used for MaxEnt: a cumulative output format; jackknife to measure the variable importance; a random test percentage = 50 %; 0.8 of the regularisation multiplier; a maximum number of background sampling points = 10,000; a replicate number = 100; a replicated run type = cross-validate; and a maximum number of iterations = 5000. Regularisation overcomes the risk of overfitting in MaxEnt. If the selected multiplier is very small (e.g., 0.01), there is great risk of overfitting and an increase in the model complexity, which affects many features in the model (e.g., linear, quadratic, and product, among others [67, 68]). By contrast, a multiplier that is high (e.g., 3) restricts the number of features included in the model too much. Empirically, we found that a multiplier of 0.8 works well with the *O. wageneri* data to neither overfit nor excessively restrict the number of features allowed in the models.

All of the sampling points without flatfish and parasite occurrences were used for the background file in the boosted GAM and MaxEnt. However, the number of sampling sites included in these files is critical for the performance of the model, and there should be good biological reasons to choose the size of this region (called M sensu Soberón and Peterson [67]), as noted elsewhere [69]. In our case, the biological justification to include all the sampling sites in the study area with presences and absences (the full area hereafter) up to a 3571 m depth and a region including only the sampling sites with depths of 1500 m or above (henceforth called the polygon area) was that helminth parasites have been reported up to depths of 5000 m [70, 71]. With respect to *S. gunteri*, the Mexican expeditions in the Gulf of Mexico searching for flatfishes have been unable to explore waters deeper than 200 m [47]; consequently, there is no reason to deny the possibility that *S. gunteri* or other fishes infected with *O. wageneri* may be detected in deeper waters in the near future.

### Evaluation of model performance for the boosted GAM and MaxEnt

It is important to bear in mind that in all boosted models (and all models that result from penalised/regularised regression), there is no p-value or significance test. To fairly evaluate the performance of both the boosted GAM and MaxEnt models, we used the following same statistical tests for both methods: Cohen’s kappa, AUC (area under the curve, also known as receiver operating characteristic curves or ROC curves) and pROC curves (partial receiver operating characteristic curves). All of these tests are based on a confusion matrix and depend on the departure from ideal scores for true positive and true negative values. Cohen’s kappa attempts to correct the degree of agreement by subtracting the portion of the counts that may be attributed to chance [72]. This coefficient ranges from -1 (total disagreement) through 0 (random classification) to 1 (total agreement). The AUC method measures the capacity of a model to determine both when a species is present and when it is absent [73]. The axes of the graph representing the AUC are 1 − specificity or the false positive rate on the X-axis and 1 − commission rate, the sensitivity or true positive rate on the Y-axis [65, 74]. The AUC graph ranges from 0 to 1 on both axes, with a 45° diagonal line between [0,0] and [1,1]. The values below this line indicate a performance no better than random, whereas values above the diagonal line between 0.7 and 0.8 are considered useful, and values >0.8 are considered excellent [75]. The foundation for the proposal of the pROC method is that the AUC method incorrectly assumes that 1 − specificity (the X-axis) spans the entire range [0,1], even when the model predictions may not span that whole range. Thus, Peterson et al. [74] proposed that changes to the AUC curves were required to generate partial ROC curves that span only a subset of the full spectrum of areal predictions. This proposal implied a change in the name of the X-axis to “proportion of area predicted present” and the assumption that the model only functions for part of the areal predictions, accepting at least a 10 % not possible to quantify on that axis [76, 77]. Thus, even when all of our dependent and independent variables were obtained in situ and within a very narrow temporal window, we do recognise the need to consider the potential effect of sampling error on the performance of the ENM models [69, 74, 78, 79]. This sampling error occurs because the spatial predictions of the ENM models present omission errors known as false negatives (omitting known distributional area) and commission errors known as false positives (the inclusion of unsuitable areas for the species distribution in the prediction) [74]. Thus, we considered a commission error (E) of 10 % for both the polygon and full area models for *O. wageneri*. Instead of using the procedure suggested by Peterson [74] and Barve [80] for the calculation of pROC curves, we used the pROC package of Robin [81]. This pROC package can be found at http://cran.r-project.org/web/packages/pROC/pROC.pdf. As mentioned above, we used the R package “dismo” to simultaneously compare the performance of different types of ENMs, including the boosted GAMs and MaxEnt (http://cran.r-project.org/web/packages/dismo/dismo.pdf [82]), while producing its own pROC curves. Finally, we compared the partial ROC curves of each model using a bootstrap test, as suggested by Pepe et al. [83], to evaluate differences in the performance of the models. Regarding the relevance of the independent variables, the mboost package has the capacity to compute bootstrap estimates to undertake a cross-validation to prevent overfitting [57]. This cross-validation involves a variable selection process that provides the frequency at which each variable is selected during the bootstrap process. Then, we used these frequencies as proxies for the importance of each of the variables within the model.

### Environmental variable layers

We interpolated each variable to build layers that were used to predict the probability of occurrence of *O. wageneri* from the models fitted (i.e., the boosted GAMs and MaxEnt). The interpolation was performed with ordinary kriging. For this procedure, we built a grid encompassing the full area and another grid for the polygon area for the dependent and independent variables.

## Results

A total of 7143 *O. wageneri* were collected from 29 out of the 162 (18 %) sampling sites. *O. wageneri* infected 163 out of 194 shoal flounders, *Syacium gunteri*. The overall prevalence, mean abundance and mean intensity of *O. wageneri* at the 29 sampling sites where the species was present were 84 %, 36 ± 45, and 44 ± 47, respectively. Across all the sampling points, the standard length and weight of the flatfishes were 12 ± 3 cm and 38 ± 45 g, respectively. The prevalence and mean intensity (± standard deviation) of *O. wageneri* per sampling site and the environmental and spatial variables selected by the boosting algorithm for the generalised additive models (boosted GAM) and MaxEnt models are shown in Tables 1 and 2, respectively. The mean abundance values of *O. wageneri* were transformed to presence/absence values as explained in the methodology section.

**Table 1:** Geographic position of the sites in the southern Gulf of Mexico, where sediment, water, shoal flounders *Syacium gunteri* and cestodes *Oncomegas wageneri* were collected

Site	Latitude	Longitude	Number of fish collected	Mean standard length of *S. gunteri* ± SD	Infection parameters of *O. wageneri*	
	DD	DD		cm	%	MA ± SD
1	-91.87749	19.063737	6	11.52 ± 0.32	100	97 ± 51
2	-92.924406	18.558687	10	10.41 ± 1.18	100	81 ± 35
3	-92.657425	18.691936	7	10.70 ± 1.42	43	19 ± 28
4	-92.460664	18.711013	4	8.05 ± 1.41	75	10 ± 9
5	-92.50014	19.187617	10	9.01 ± 0.89	80	29 ± 22
6	-91.500028	19.374545	1	23.30	100	13
7	-93.000353	18.753978	11	12.48 ± 1.23	89	73 ± 60
8	-95.501	19.001317	10	10.95 ± 1.17	80	8 ± 6
9	-92.500832	18.999193	9	12.47 ± 2.06	100	20 ± 22
10	-92.006873	18.999577	3	16.87 ± 4.59	67	62 ± 9
11	-91.501037	19.500352	4	23.38 ± 5.54	75	11 ± 13
12	-92.00304	18.937508	6	12.28 ± 0.47	100	94 ± 68
13	-92.375937	18.999565	6	11.18 ± 0.46	100	39 ± 14
14	-95.999833	19.501753	10	12.59 ± 1.31	60	61 ± 108
15	-94.25	18.233333	10	10.97 ± 1.15	80	13 ± 9
16	-92.15496	18.88098	9	11.00 ± 0.71	100	34 ± 15
17	-92.986852	18.483098	4	11.25 ± 0.26	100	110 ± 44
18	-93.00009	18.50345	11	11.35 ± 1.23	82	60 ± 40
19	-93.20285	18.62565	10	17.78 ± 5.62	80	22 ± 23
20	-92.81203	18.691928	11	9.81 ± 0.97	100	33 ± 27
21	-92.563643	19.126108	11	11.32 ± 1.88	90	37 ± 23
22	-92.50009	19.251617	6	13.37 ± 4.15	17	27
23	-92.18765	18.938898	4	10.74 ± 0.82	100	40 ± 46
24	-92.252252	18.772892	3	8.83 ± 2.48	100	25 ± 27
25	-92.12616	19.063458	5	14.16 ± 7.30	80	51 ± 53
26	-94.000493	18.375	6	15.13 ± 0.93	100	26 ± 30
27	-92.390441	18.68429	2	10.45 ± 5.65	100	43 ± 39
28	-92.063815	18.812852	4	10.90 ± 5.53	100	11 ± 7
29	-94.499377	18.753407	1	11.30	100	214

DD = decimal degrees, % = prevalence, MA ± SD = mean abundance ± standard deviation. See Additional file 1: Table S1 for a complete list of physicochemical parameters and contaminants from water and sediments

**Table 2:** Independent variables selected by the boosted general additive model (boosted GAM) for the full area and polygon area models

	Independent variables	Units	Full area	Polygon area
			Fr (%)	Fr(%)
1	bspatial (Lon, Lat)	DD	1.45	18.90
2	bbs (Total depth, S)	m	-	4.80
3	bbs (Temperature, W)	°C	0.09	5.40
4	bbs (Salinity, W)	UPS	0.13	0.60
5	bbs (Oxygen, W)	(mg/L)	0.17	5.00
6	bbs (Alkalinity, W)	meq/l	0.13	2.10
7	bbs (CO2, W)	mmol/l	0.13	2.10
8	bbs (Nitrate, W)	μMolar	0.21	3.00
9	bbs (Phosphate, W)	μMolar	0.09	0.70
10	bbs (Silicate, W)	μMolar	-	1.10
11	bbs(Sigma T, W)	Kg/m3	0.30	-
12	bbs(Sand, S)	%	-	0.50
13	bbs (Silt, S)	%	-	1.10
14	bbs (Clay, S)	%	0.04	-
15	bbs (Phosporus, S)	micromol/g	0.04	0.60
16	bbs (Nitrogen, S)	micromol/g	-	1.30
17	bbs (PAHL, S)	μg/g	0.47	5.70
18	bbs (PAHH, S)	μg/g	94.70	6.90
19	bbs (Aliphatic PAHs, S)	μg/g	-	0.60
20	bbs (PCNM2)	DD	0.30	24.00
21	bbs (PCNM21)	DD	0.47	3.70
22	bbs (PCNM58)	DD	0.68	11.90

Fr (%) is the frequency at which each variable is selected during the bootstrapping, and used as proxy of the importance of each of the variables (expressed as percentage) within the model. bbs = base-learner based on B-splines, bspatial = spatial base-learner on geographical coordinates. W = water, S = sediment; PAHL and PAHH are polyaromatic hydrocarbons of low and high molecular weight respectively; PCNM2, PCNM21 and PCNM58 are the spatial variables of the principal coordinates of neighbour matrices (PCNM) analysis, acting at 2, 21 and 58 km respectively

### *Oncomegas wageneri* boosted GAM and MaxEnt

The number of sampling sites where *O. wageneri* were present and their mean abundance values per sampling site are shown in Fig. 1a and b. Figure 1c shows the probability of *O. wageneri* occurrence for the full area, which had a strong statistical association with high molecular weight polyaromatic hydrocarbons (PAHH) (selected by the bootstrap analysis with a 95 % frequency, Table 2). By contrast, all the remaining spatial and nutrient variables showed a minor contribution (5 %) to the explained variability (Table 2). Thus, for the full area, the final model was: Probability of the occurrence of *O. wageneri* ~ bbs (PAHH) + all other 16 variables in the full area column in Table 2. The boosted GAM for *O. wageneri* across the full area (Fig. 1c) shows that the probability of the occurrence for this parasite was high in the region between Cayo Arcas and the Coatzacoalcos River mouth (orange and yellow zones), whereas the continental shelf of the Yucatan Peninsula and the oceanic region in the middle of the Gulf of Mexico showed a very low occurrence probability for this parasite. Thus, the probability of *O. wageneri* occurrence based on the boosted GAM for the full area (Fig. 1c) closely resembles the actual spatial distribution of *O. wageneri* in the study area (Fig. 1a and b).

For the polygon area (Fig. 1d), the probability of *O. wageneri* occurrence was similar to the one using the full area (Fig. 1c). However, the number of independent variables differed between the models with 17 for the full area and 20 for the polygon area (Table 2). For the polygon area, the most important component of the model was related to 3 spatial variables (55 % frequency all together), followed by the PAHH (7 % frequency). Thus, for the polygon area, the final model was: Probability of the occurrence of *O. wageneri* ~ bspatial (Lon, Lat) + bbs (PCNM2) + bbs (PCNM58) + bbs (PAHH) + all other 16 variables in the polygon area column in Table 2. The MaxEnt model for *O. wageneri* (Fig. 1e and f) for the full area and for the polygon area poorly predicted the probability of *O. wageneri* occurrence (Fig. 1a). The performance statistics (kappa, AUC and pROC) from the boosted GAM for the full area and the polygon area for *O. wageneri* were all above 0.8 (Table 3). By contrast, all the performance statistics in the MaxEnt models for *O. wageneri* for the full area and the polygon area were below 0.8 (Table 3).

**Table 3:** Performance statistics of the boosted general additive model (boosted GAM) and MaxEnt models for *Oncomegas wageneri* for the full area and the polygon area

	Boosted GAM		MaxEnt	
	Full area	Polygon area	Full area	Polygon area
Kappa	0.823	0.823	0.577	0.434
AUC	0.970	0.959	0.679	0.466
pROC	0.837	0.833	0.737	0.649
	(0.722-0.941)^a^	(0.725-0.949)	(0.561-0.912)	(0.474-1.000)

Kappa = Cohen’s kappa, AUC = area under the curve and pROC = partial receiver operating characteristic curve. ^a^ The values in brackets were the ranges obtained by bootstrapping

## Discussion

The main hypothesis tested in this paper was that because *O. wageneri* has transmission stages and intermediate hosts that are exposed to a polluted environment, the probability of the occurrence of this parasite should reflect the environmental conditions experienced at the seascape level. Our results suggest that this pattern occurred. *O. wageneri* was widely distributed near the coast (Fig. 1c) and was very much influenced, not only by the distribution of the shoal flounder, but also by the PAHH and nutrients present in the shallow waters of the coastal zone (Fig. 1c; Table 2). However, before undertaking a detailed interpretation of the statistical relationships between the dependent and independent variables in the boosted GAMs and the MaxEnt models for this parasite species it is necessary to address two issues: the effect of the size of the background area (full or polygon areas) and the marked differences in the values of the performance statistics between the ENMs for *O. wageneri* (Table 3).

### The background area and the performance of the ENMs

For *O. wageneri*, the size of the background area (or accessible area sensu Barve et al. [69]) considered (full and polygon areas) did not affect the performance of the boosted GAM (Table 3) but did influence the number and identity of the environmental and spatial variables associated with the probability of the occurrence of this parasite (Table 2). By contrast, the background area considered (either full or polygon areas) was not relevant to the poor performance of the MaxEnt models for *O. wageneri*, which was a rather surprising result (Table 3). It is possible that the relatively low number of sampling sites where *O. wageneri* occurred in our study (29 (18 %) out of 162 sampling sites) affected the performance of the MaxEnt models. This is a very unusual result because the MaxEnt method is normally very reliable even with few occurrences [65]. Even by reducing the size of the background zone from the full area to the polygon area to resemble the size of the actual *O. wageneri* distribution area, there was no improvement in the performance statistics (Table 3). As an extra test, we reduced the size of the background to the size of the *O. wageneri* occurrence region, as suggested by Phillips et al. [84], but that strategy also failed to improve the values of the performance statistics. Thus, it is possible that the boosted GAMs would be a better choice for the analysis of problematic data sets of this type. Certainly, it would be desirable (but expensive) to perform more sampling in the region where *O. wageneri* is present for the construction of a comparable background region to obtain a comparable number of sampling sites with absences, as suggested by Phillips et al. [84], to correct for the potential effect of a geographical bias.

Considering the specific case of the boosted GAM for *O. wageneri* sampled in the full area, the performance of the model was excellent (Table 3) because all the values of the Kappa, AUC and pROC performance statistics were between 0.8 and 0.9 [75]. The model included 17 variables; the most influential of which was the PAHH (frequency = 95 %) followed by a minor contribution from a combination of nutrients, the PAHL and the PCNM spatial variables (frequency = 5 %) (Table 2). Considering the polygon area, the performance of the model was also excellent. However, the model was increased to 20 variables; the most important of which were the three spatial ones (frequency = 55 % all together) followed by a combination of environmental and spatial variables with minor frequency values (Table 2). Considering the MaxEnt models for *O. wageneri* sampled in the full area and the polygon area, the values of the Kappa and pROC performance statistics were between 0.4 and 0.7. Because these values indicate a very poor performance [75], no further interpretation of the MaxEnt models or the environmental variables associated with these models was considered necessary. Applying the criterion of parsimony for choosing the best *O. wageneri* model for interpretation, we selected the boosted GAM with a smaller number of variables; that is, we selected the full area model shown in Fig. 1c over that of the polygon area because, despite similarly good values for their performance statistics, the model for the full area had fewer variables (Table 2). Thus, the rest of the discussion concentrates on the interpretation and explanation of the patterns obtained with the boosted GAM for *O. wageneri* sampled in the full area (Fig. 1c).

### Values of the infection parameters of *O. wageneri* in the full area

The values of the prevalence and mean abundance of *O. wageneri* infecting the shoal flounder in the full area (Table 1) were high and similar to those obtained for the Mexican flounder *Cyclopsetta chittendeni* [85] (prevalence range 17-100 %; mean abundance range: 8 ± 6-110 ± 44). These results suggest that the transmission of the larval forms of *O. wageneri* in the southern Gulf of Mexico is high. However, even though the larvae of *O. wageneri* have been reported by several authors to infect fish in the Gulf of Mexico [35, 51, 86], none of these authors provided data on the prevalence or mean abundance of this parasite for other zones in the Gulf of Mexico. Thus, it is difficult to know if the high values of the infection parameters of *O. wageneri* in *S. gunteri* that we found could be similar to those in other regions of the Gulf of Mexico.

### Statistical relationships in the boosted GAM for *O. wageneri*

The PAHH variable had the highest frequency (95 %) in the boosted GAM for the full area *O. wageneri* model (Table 2), which suggests that both the shoal flounder and *O. wageneri* have been chronically exposed to these pollutants. The PAHL were present in the *O. wageneri* models but with minor frequency (Table 2). One of the most common indices to determine the main source of PAH is the PAHL/PAHH ratio. If this ratio is <1, the most likely origin is pyrolytic (incomplete combustion of organic matter - combustion of fossil fuel, vehicular engine combustion, smelting, waste incinerators, forest fires and coal combustion), whereas values >1 indicate a petrogenic origin (unburned petroleum and its products - gasoline, kerosene, diesel, lubricating oil and asphalt) [87, 88]. In our data set, only 14 of the 162 sampling sites had PAHL/PAHH ratios >1 (See Additional file 1: Table S1). Thus, most of the PAHs to which the flatfishes and their parasites had been exposed were pyrolytic. Regarding the potential toxicological effects of PAHL and PAHH, none of these compounds exceeded the probable effect level (PEL) established for marine and estuarine sediment quality [89], but several (e.g., benzo[a]pyrene) are considered carcinogenic [90]. Therefore, whether these compounds have a direct effect on *O. wageneri*, the shoal flounder or the definitive hosts (rays) remains an open question.

Another question is why were the PAHH selected preferentially by the *O. wageneri* boosted GAM? It is likely that the presence of these compounds together with the presence of the PAHL, N and P carried from the continent through the river discharge into the marine sediments enhances the growth of hydrocarbonoclastic bacteria; these are common, free-living bacteria in marine environments and include, for example, certain species of the genera *Bacillus*, *Pseudomonas* and *Halomonas* that feed on these compounds [91, 92]. These bacteria feed easily on the PAHL, but it takes them a long time to decompose the PAHH, if they do at all [93], which in turn could be a potential explanation for the persistent presence of the PAHH in the boosted GAM model. In any case, an increase in the number of colonies of these bacteria would in turn enhance primary and secondary productivity in the area with a consequent increase in the number of intermediate hosts, which has been suggested for regions affected by oil spills, such as the Prestige spill [16, 17]. The above-mentioned provision of nutrients and the PAHH from the continent into the coastal zones (up to 200 m depth) has been widely documented [33] for the marine region from Terminos Lagoon to the Coatzacoalcos River zone. However, based on the available data, it is difficult to infer what deleterious effects are induced by the PAHH (and other pollutants) on the transmission stages (coracidia, plerocercoids), on the first and second intermediate hosts (copepods and shrimps, respectively), on the shoal flounder (acting as paratenic or third intermediate host) or even on the definitive host (stingrays). Thus, experimental exposure of the components of the life cycle of *O. wageneri* (hosts and transmission stages) will be necessary to clarify the relative effects of these persistent organic pollutants (POPs) because previous work has stressed their potential deleterious effects on the life cycles of marine parasites [94, 95].

The frequency of the spatial component in the boosted GAM of *O. wageneri* (summing the bspatial and the PCNMs variability) was very small (1.45 %) (Table 2). This result suggests that the spatial autocorrelation had a small effect on the models for *O. wageneri*, which most likely occurred because the shoal flounder also likely had a fairly high site fidelity, meaning that these individuals do not move large distances as the pelagic fishes do. These flounders (e.g., *Syacium gunteri*) do not enter the coastal lagoons but complete their life cycle in a relatively narrow region with small migrations from coastal to deeper oceanic waters (~100 m depth) for feeding and reproduction [47, 96]. For these flatfishes and their parasites, the environmental variables that occur at the local level (in the very same place where they live) were far more important than the environmental variables acting at larger spatial scales. This interpretation agrees with that of Hothorn et al. [63], who suggested that the mobility of the species under study has a large influence on the relevance of the spatial component in the boosted GAMs. These researchers found that in highly vagile birds, the spatial component was very important; however, for dragonflies with a low spatial range, this component was largely irrelevant.

## Conclusions

Our results have led us to two conclusions. One involves a methodological issue, and the other concerns a biological issue. The conclusion regarding methodology was that the boosted GAMs applied to the full area were excellent for describing the probability of the occurrence of *O. wageneri* in the southern Gulf of Mexico, as suggested by the values of the Kappa, AUC and pROC performance statistics (Table 3) and based on the scale proposed by Hosmer and Lemeshow [75]. By contrast, the models produced by the MaxEnt performed very poorly for *O. wageneri*. Furthermore, decreasing the background zone to the polygon area so that it was the same size as the parasite occurrence area, which included only the sample sites shallower than 1500 m or even smaller, (as suggested by Phillips et al. [84]), did not improve performance. Thus, the conclusion at this time is that the MaxEnt is not a good tool to describe the probability of the occurrence of *O. wageneri* infecting the shoal flounder in the southern Gulf of Mexico.

With respect to our biology-related conclusion, 10 out of the 14 sampling sites with PAHL/PAHH ratios values >1 had significantly lower values for the prevalence of *O. wageneri* than those sites with values < 1 (Fisher’s exact test; *p* <0.0001). In the case of the mean abundance, it was not possible to calculate whether there were differences between sites with PAHL/PAHH ratio values >1 or < 1 because there were only four sites with PAHL/PAHH ratio values >1 and few individual fish for comparison (Additional file 1: Table S1). Altogether, our results suggest relatively high prevalence values of *O. wageneri* for sites with PAHL/PAHH ratios values between 0 and 1.89 (Additional file 1: Table S1). The origin of these PAHH is most likely petrogenic, but the problem is apparently still not extreme, judging by the high prevalence values of *O. wageneri* in the present study (Table 1 and Additional file 1: Table S1). The 10 sites with PAHL/PAHH ratio values > 1 (range 1.90 to 3.13), had neither fish nor parasites. We concluded that sites with a PAHL/PAHH ratio value up to 1.89 had an enhanced transmission based on the high values of the prevalence of *O. wageneri* (Additional file 1: Table S1), while the sites with PAHL/PAHH ratio values ≥ 1.90 were harmful for both the fish and the parasites because, apparently, they are not able to persist in those sites (Additional file 1: Table S1), which in turn negatively affects their probability of occurrence.

## Additional file

Additional file 1: Table S1. Geographic positions, the number of shoal flounders (*Syacium gunteri*) collected and the environmental variables recorded for each sampling site. The variables from Depth to Sigma-T were obtained from water, and those from Carbon % to Total hydro from sediments. PAHL and PAHH are polyaromatic hydrocarbons of low and high molecular weight respectively; Oxygen sat is oxygen saturation; pHW and pHS are pH values for water and sediment respectively; UCM is unresolved complex mixture and Total hydro is the sum of Total PAHs, Aliphatics and UCM. (XLSX 75 kb)
